# *Oryzias curvinotus* in Sanya Does Not Contain the Male Sex-Determining Gene *dmy*

**DOI:** 10.3390/ani11051327

**Published:** 2021-05-06

**Authors:** Zhongdian Dong, Xueyou Li, Zebin Yao, Chun Wang, Yusong Guo, Qian Wang, Changwei Shao, Zhongduo Wang

**Affiliations:** 1 Guangdong South China Sea Key Laboratory of Aquaculture for Aquatic Economic Animals, Fisheries College, Guangdong Ocean University, Zhanjiang 524025, China; lxycjhj@163.com (X.L.); yaozebin@stu.gdou.edu.cn (Z.Y.); chunwang_2019@163.com (C.W.); ysguo@gdou.edu.cn (Y.G.); 2 Guangdong Provincial Engineering Laboratory for Mariculture Organism Breeding, Fisheries College, Guangdong Ocean University, Zhanjiang 524025, China; 3 Guangdong Provincial Key Laboratory of Pathogenic Biology and Epidemiology for Aquatic Economic Animals, Fisheries College, Guangdong Ocean University, Zhanjiang 524025, China; 4 Yellow Sea Fisheries Research Institute, Chinese Academy of Fisheries Sciences, Qingdao 266071, China; wangqian2014@ysfri.ac.cn (Q.W.); shaochangwei303@163.com (C.S.); 5 State Laboratory of Developmental Biology of Freshwater Fish, College of Life Sciences, Hunan Normal University School, Changsha 410081, China

**Keywords:** *Oryzias curvinotus*, *dmy*, sex determining gene, RNA-Seq

## Abstract

**Simple Summary:**

*dmy* is considered to be the male-determining gene in Japanese medaka (*Oryzias latipes*) and Hainan medaka *(Oryzias. curvinotus*)*,* both of which have the XX/XY sex-determination system. Here, we found a group of medaka in the Sanya River (named SY-medaka) and confirmed that SY-medaka belongs to *O. curvinotus* by morphological characteristics and mitochondrial phylogenetic analysis. Through genetic sex identification, genome re-sequencing and gonadal transcriptome analysis, it was preliminary confirmed that SY-medaka did not contain *dmy*. Our results provide a basis for further studies of the mechanism underlying sex determination in *Oryzias* and functional genomics and reproduction biology in *O. curvinotus*.

**Abstract:**

Hainan medaka (*Oryzias curvinotus*) is distributed in the coastal waters of the South China Sea and is able to adapt to a wide range of salinities. In this study, we characterized *O. curvinotus* in Sanya River (SY-medaka), which lacks *dmy* (a male sex-determining gene in *O. latipes* and *O. curvinotus*). In a comparison of SY-medaka and Gaoqiao medaka (GQ-medaka), the morphological difference between the two populations does not reach the subspecies level and they can be considered two geographic populations of *O. curvinotus*. A mitochondrial cytochrome oxidase subunit I (CoI) sequence alignment showed that the sequence identities between SY-medaka and other geographic populations of *O. curvinotus* are as high as 95%. A phylogenetic analysis of the mitochondrial genome also indicated that SY-medaka belongs to *O. curvinotus*. Molecular marker-based genetic sex assays and whole genome re-sequencing showed that SY-medaka does not contain *dmy*. Further, in RNA-Seq analyses of the testis and ovaries of sexually mature SY-medaka, *dmy* expression was not detected. We speculate that high temperatures resulted in the loss of *dmy* in SY-medaka during evolution, or the lineage has another sex-determining gene. This study provides a valuable dataset for elucidating the mechanism underlying sex determination in *Oryzias* genus and advances research on functional genomics or reproduction biology in *O. curvinotus*.

## 1. Introduction

Hainan medaka (*Oryzias curvinotus*) has recently been reported along the coast of the South Sea [1,2]. *O. curvinotus* is able to survive in both hyperosmotic and hypoosmotic environments and is an emerging model for analyses of osmotic adaptation as well as for marine environmental and toxicological studies [1]. Previous studies have shown that *O. curvinotus* employs a XX/XY sex chromosome system and has the sex-determining gene *dmy* [3,4,5]. Accordingly, it has the potential to become a model for studies of sex determination and differentiation in marine fish.

Various sex-determining genes in fish have been identified, including *dmy* (Y-specific DM-domain) in *Oryzias latipes* and *O. curvinotus* [3,4,5,6], *sdy* (sexually dimorphic on the Y chromosome) in *Oncorhynchus mykiss* [7], *amhr*2 (anti-Mullerian hormone receptor type II) in *Takifugu rubripes* [8], *amhy* in *Odontesthes hatcheri* [9], *gsdf* (gonadal soma-derived growth factor on the Y chromosome) in *Oryzias luzonensis* [10], and *dmrt*1 (Doublesex and mab-3 related transcription factor 1) in *Cynoglossus semilaevis* [11,12]. However, the sex-determining genes in most fish are still unclear. RNA-seq is a rapid method to obtain gene expression data in the absence of sequenced genomes [13]. This approach has been used to identify sex-related genes and for transcriptome profiling in relation to gonadal development and gametogenesis in fish, including *Oreochromis niloticus* [14], *Scatophagus argus* [15], and *Sillago sihama* [16]. We found a population of medaka (named SY-medaka) in the Sanya River, southern Hainan province, which is suspected to be *O. curvinotus*.

Morphological divergences analysis is the traditional and one of the most basic methods for the study of fish taxonomy [17,18,19]. Mitochondrial genes are highly conserved and can be used for species classification [20]. Phylogenetic analyses based on mitochondrial sequences can be used to identify genetic relationships among species [21,22]. In this study, we collected SY-medaka and determined whether it belongs to *O. curvinotus* by morphological and mitochondrial genome analyses. We also evaluated sex-specific molecular markers in SY-medaka and compared the transcriptomes of the testis and the ovaries in the population for the first time.

## 2. Materials and Methods

### 2.1. Ethics Statement

The work described in this article has been carried out in compliance with committee at the Guangdong Ocean University. All samples were obtained under MS222 anesthesia.

### 2.2. Collection of Fish and Samples

Wild sexually mature medaka (body length 29.25 ± 1.5 mm; body weight 0.27 ± 0.03 g, salinity, 30 ppt) were collected from Sanya River (named SY-medaka), Hainan Province, China (18°14′29.50″ N, 109°30′29.76″ E). The wild individuals were desalinated in the laboratory, cultured in fresh water for 30 days, and anesthetized by soaking with MS222 (20 μg/L) for anatomic dissection. The physiological sex of SY-medaka was determined by anatomical gonadal type, testis and ovaries were dissected and frozen in liquid nitrogen for RNA extraction, and fins were placed in alcohol for DNA extraction.

### 2.3. Morphological Traits and Molecular Species Identification

Nineteen morphological traits of SY-medaka and GQ-medaka (medaka collected from the National Mangrove Nature Reserve in Gaoqiao, Guangdong Province, China) (21°36′24” N, 109°47′8” E) [23] were compared and analyzed (Table 1). Covariance analysis was used to compare the morphology of the two populations of medaka. In the analysis of covariance, body length was taken as the covariable, and other measurable traits were corrected and compared, and then unified analysis of variance was conducted. The coefficient difference (Cd) was calculated by the following formula: Cd = (M1 − M2)/(S1 + S2). M1, M2, S1, and S2 respectively correspond to the mean value and standard deviation (sd) of the trait indexes of the two populations. A total of 50 SY-medaka and 41 GQ-medaka were counted and measured. Measurements were performed following the methods of Zhang et al. [24]. SPSS18.0 (IBM, New York, NY, USA) was used to perform one-way analysis of variance.

Genomic DNA was extracted from each sample using the TIANamp Marine Animals DNA Kit (TIANGEN, Beijing, China) according the instructions. DNAs from 10 females and 10 males of SY-medaka were used to detect the mitochondrial cytochrome oxidase subunit I (COI)sequence for species identification. COI-F/COI-R primers (Table S1) were used for PCR amplification, and the PCR program was as follows: 95 °C for 5 min; 35 cycles of 95 °C for 30 s, 50 °C for 30 s, and 72 °C for 1 min; then 72 °C for 7 min for extension. PCR products were sent to Genewiz Company (Suzhou, China) for sequencing. DNAs from 100 females and 130 males of SY-medaka were used for genetic sex determination, using GQ-medaka as a positive control. One pair of primers, Ocsex-F/Ocsex-R (Table S1), which can amplify different size fragments of *dmrt*1 and *dmy* in *O. curvinotus,* was used to detect the genetic sex of SY-medaka [25]. The reaction conditions were as follows: 95 °C for 5 min; 35 cycles of 95 °C for 30 s, 60 °C for 30 s, and 72 °C for 1 min; then 72 °C for 7 min for extension. PCR products were examined by electrophoresis on 1.5% agarose gels. Using this method, if a PCR product yielded a single band by electrophoresis, the individual was identified as a genetic female (XX); if a double band was obtained, the individual contained *dmy* and was a genetic male (XY) [25].

### 2.4. Genome Re-Sequencing

Fifteen SY-medaka and fifteen GQ-medaka individuals were used for genome re-sequencing. The genomic DNA was extracted with a total amount of 1.5 µg per sample for library construction. Sequencing libraries were generated using Truseq Nano DNA HT Sample preparation Kit (Illumina, California, CA, USA) following manufacturer’s recommendations, and index codes were added to attribute sequences to each sample. Briefly, the DNA sample was fragmented by sonication to a size of 350 bp, then DNA fragments were end polished, A-tailed, and ligated with the full-length adapter for Illumina sequencing with further PCR amplification. At last, PCR products were purified and libraries were analyzed for size distribution by Agilent2100 Bioanalyzer and quantified using real-time PCR. Whole genomes of 30 samples were sequenced based on the Illumina Hiseq PE150 platform. The Hiseq sequencing was converted to raw data by Base Calling, and the lower-quality reads (Reads with ≥10% unidentified nucleotides (N); >10 nt aligned to the adaptor, allowing ≤10% mismatches; >50% bases having phred quality < 5; and putative PCR duplicates generated in the library construction process) were removed to obtain clean data. Clean data was compared to the reference genome by BWA software (parameter: mem-t 4-k 32-M) [26], and duplicates were removed by SamTools (parameter: rMDUP) [27].

### 2.5. RNA Isolation, cDNA Library Preparation, and Sequencing

To further determine whether SY-medaka expresses *dmy*, RNA-Seq was performed using the testes and ovaries of SY-medaka. The gonadal development of SY-medaka was analyzed by a histological analysis of sections. For males, testes from four individuals were pooled to obtain one sample. For females, the ovaries of one individual were used as one sample. One sample was used to construct one cDNA library, and eventually three testis libraries and three ovary libraries were obtained. Total RNA isolation and cDNA library preparation were completed by BGI (Beijing, China). For the details of the procedure, refer to Lin et al. [28]. The library was sequenced using the BGISEQ-500 sequencing platform (MGI Tech Co., Ltd., Shenzhen, China) and paired-end 100-bp reads were generated. All raw data have been submitted to the CNGB Nucleotide Sequence Archive (CNSA) under the accession number CNP0000963.

### 2.6. De Novo Assembly and Functional Annotation

Clean data were obtained by removing low-quality reads (i.e., reads with more than 20% of the bases having a Phred score lower than 15), reads with adaptors, and reads with unknown bases (N bases more than 5%). Those clean reads were de novo assembled using Trinity (v2.0.6), and gene family clustering was performed using Tgicl (v2.0.6) to obtain the final unigenes for functional annotation. BLASTn (v2.2.23, http://blast.ncbi.nlm.nih.gov/Blast.cgi (accessed on 8 October 2018)) with an E-value threshold of 1 × 10^−5^ was used to query the unigenes against Nt (NCBI non-redundant nucleotide sequences). Diamond (v0.8.31, https://github.com/bbuchfink/diamond (accessed on 14 August 2018)) with an E-value cut-off of 1 × 10^−5^ was used to query the unigenes against the NR (NCBI non-redundant protein databases), KOG (eukaryotic orthologous group database), KEGG (Kyoto Encyclopedia of Genes database), and Swiss-Prot protein databases. Gene ontology (GO) annotation of unigenes was performed using Blast2GO based on the National Center for Biotechnology Information (NCBI) NR database annotation results [29].

### 2.7. Differential Gene Expression Analysis and Quantitative Real-Time PCR (qPCR) Validation

Clean reads were mapped to unigenes using Bowtie2 [30], and the gene expression level was calculated using RNA-Seq by Expectation Maximization (RSEM) [31]. The fragments per kilobase per million reads (FPKM) method was used to quantify gene expression levels. The differentially expressed genes (DEGs) were detected using NOIseq as described by Tarazona et al. [32]. Unigenes with Fold Change ≥ 2.00 and Probability ≥ 0.9 between the testes and ovaries were considered DEGs. Twenty genes were selected to verify the accuracy of the sequence assembly by PCR and sequencing, and 20 were used for the validation of the transcript expression profile by quantitative real-time PCR (qPCR). qPCR primers (Table S1) were designed according to transcriptomic results, and PCR amplification and clone sequencing were performed with each pair of qPCR primers. qPCR was performed using the Roche LightCycler 96 system (Roche, Forrentrasse, Switzerland). qPCR amplification conditions were as follows: 180 s at 95 °C for pre-incubation, followed by 40 cycles at 95 °C (15 s), 60 °C (15 s), and 72 °C (30 s). The combination of eef1b and rps4x was used as the internal reference, and three technical repetitions were used for each sample [23]. The transcript abundance of each gene relative to the corresponding solvent control was log_2_-transformed according to the ^2-ΔΔCt^ method [33].

## 3. Results

### 3.1. Morphological and Molecular Analyses of SY-Medaka

We compared countable and measurable traits of SY-medaka and GQ-medaka by one-way analysis of variance. Except for the significant difference in caudal fin ray counts between the SY-medaka (19.31 ± 1.02) and the GQ-medaka (18.51 ± 0.69), there were no differences in the other four traits between the two groups (Figure S1, Table 1). For measurable traits, total length, head length, tip of the snout to dorsal fin, caudal peduncle depth, length of the dorsal fin, length of the base of the anal fin, and length of the pectoral fin differed significantly between groups (*p* < 0.05). The coefficient of variation between the two groups was highest for the length of the pectoral fin at 0.58 (Table 1).

**Figure 1:**
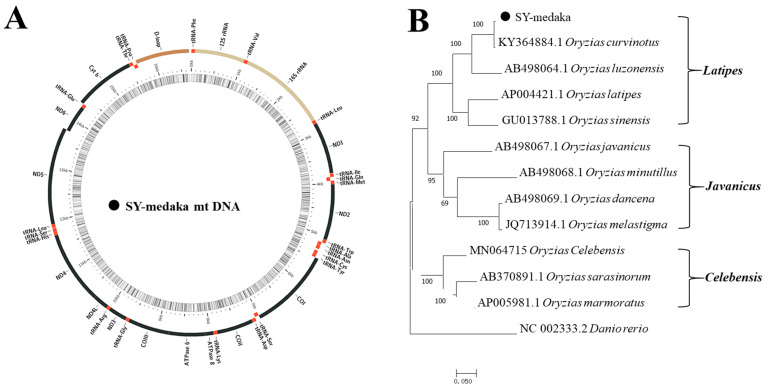
Structure of the mitochondrial genome of SY-medaka (medaka in the Sanya River) (**A**) and phylogenetic tree analysis of the mitochondrial sequence of *Oryzias* (**B**). The phylogenetic tree was constructed using MEGA 6.0 with the maximum likelihood method. Black point indicates SY-medaka.

COI of SY-medaka was cloned and sequenced (Table S2). The sequence identity between SY-medaka and GQ-medaka was as high as 95% (data not shown). Furthermore, a 16,667-bp mitochondrial genome was assembled from RNA-seq data, including 13 protein-coding genes and 22 tRNA genes (Figure 1A). Sequence alignment by BLAST (https://blast.ncbi.nlm.nih.gov/Blast.cgi) showed that SY-medaka has maximum identities with *O*. *curvinotus* (99.33%), followed by *O. luzonensis* (88.25%) and *O. latipes* (83.89%). An ML phylogenetic tree was constructed based on the complete mitochondrial genome sequence of 11 medaka fishes, with zebrafish as the outgroup. The results showed that SY-medaka and *O. curvinotus* clustered together, and *O. luzonensis*, *O. latipes*, and *O. sinensis* formed a separate cluster (Figure 1B). Furthermore, the evolutionary divergence analysis of mitochondrial sequences among the above-mentioned fish indicated that the genetic distance between SY-medaka and *O. curvinotus* was only 0.005, while the genetic distance between SY-medaka and *O. luzonensis* was 0.095, which was similar to the genetic distance between *O. curvinotus* and *O. luzonensis* (0.094) (Table S3).

### 3.2. Genetic Sex Identification of SY-Medaka

**Figure 2:**
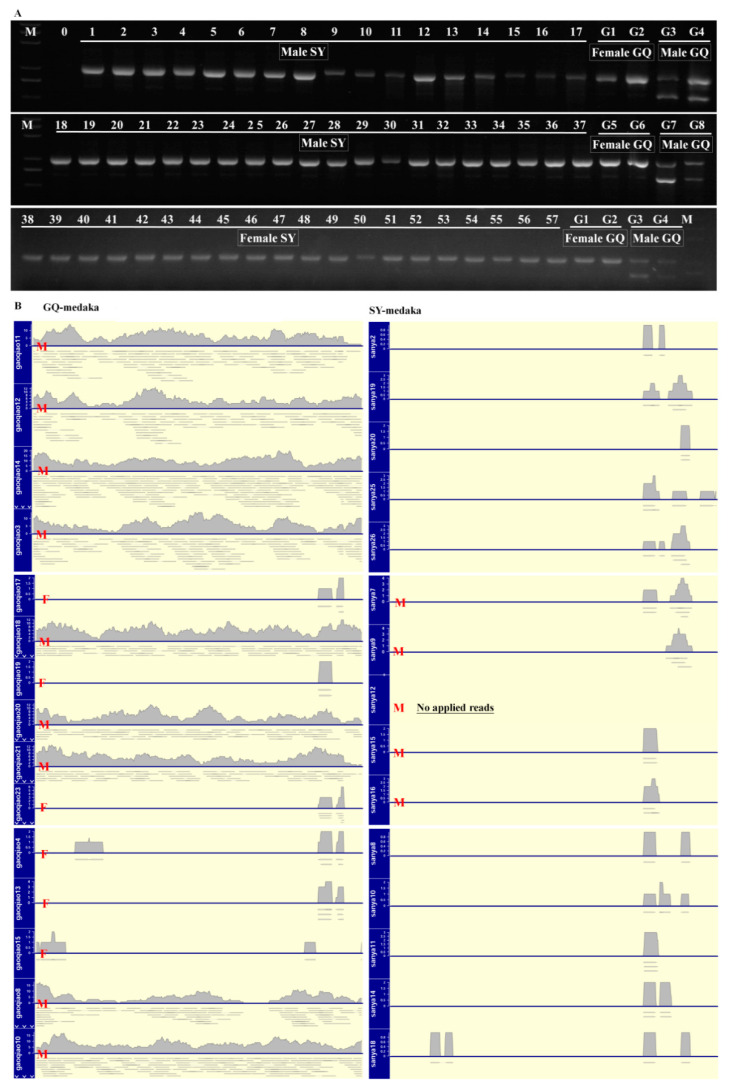
Genetic sex identification of *O. curvinotus*. (**A**) Gel electrophoresis results of PCR products amplified from the genome of *O. curvinotus* by primer Ocsex-F/Ocsex-R. Male SY-medaka (medaka in the Sanya River): 1–17 and 18–37. Male GQ-medaka (Gaoqiao medaka): G3, G4, G7, and G8. Female SY-medaka: 38–57. Female GQ-medaka: G1, G2, G5, and G6. 0 is a negative control; M is a DL 5000 DNA marker. (**B**) Coverage of *dmy* by whole genome re-sequencing reads of GQ-medaka and SY-medaka. M indicates male and F indicates female. Only five males of SY-medaka (7, 9, 12, 15, and 16) had complete sexual identification records, unfortunately the sampling records of the remaining 10 samples (six females and four males) were missing, making it impossible to pinpoint the sex of each sample.

Genetic assays were performed with the sex-specific primers Ocsex-F and -R [25]. After amplification, only one band was detected for both male and female SY-medaka. However, in GQ-medaka, females had a single band and males had two bands (Figure 2A). The *dmy* locus was not detected in any of the tested individuals of SY-medaka. Further, we analyzed re-sequencing data for 15 SY-medaka and 15 GQ-medaka. The males in the two groups showed differentiation in *dmy*. The coverage of *dmy* per male and female individual in SY-medaka was consistent and similar to that of female GQ-medaka (Figure 2B). *Dmrt*1 was selected as a control for *dmy* coverage analysis to verify the re-sequencing data was reliable, the coverage rate of *dmrt*1 in SY-medaka and GQ-medaka was almost the same, and there was no significant difference between females and males (Figure S2).

### 3.3. RNA-Seq Analysis, Unigene Annotation, and dmy Detection

The results of gonad histological section showed that the SY-medaka used in this study were sexually mature (Figure S3). A total of 36.69 Gb of data was generated using the BGISEQ-500 platform (Table S4). Using Trinity, 84,484 unigenes were assembled with an N50 length of 2384 bp, and 93.87% of unigenes (79,301) were annotated (Table S5). By searching against the NR database, 46,375 unigenes matched to known sequences from 430 species, and the top three species were *O. latipes* (35,653; 76.88%), *Larimichthys crocea* (9523; 15.46%), and *Lates calcarifer* (3210; 5.21%) (Figure S4). By searching the annotation results, *dmy* mRNA expression was not detected. The nucleic acid and amino acid sequences of *O. curvinotus dmy* (GenBank accession nos: BAC65995.1) were used to generate a local BLAST alignment of transcriptome data, and *dmy* was not included in the hits.

### 3.4. Differentially Expressed Gene Identification and qPCR Validation

**Figure 3:**
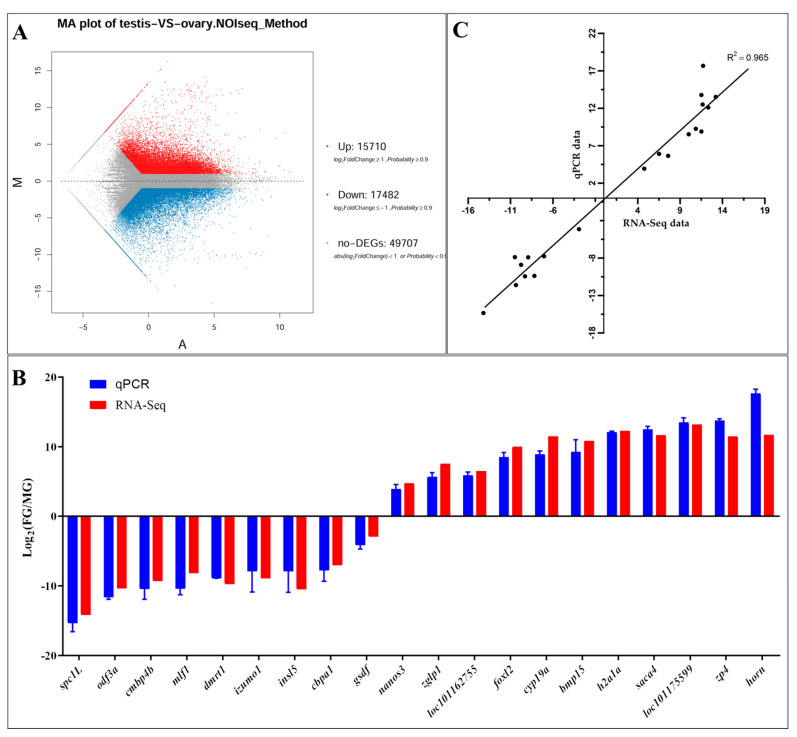
(**A**) MA plot of differentially expressed genes between the testis and ovary. (**B**) Validation of RNA-Seq data using qPCR. The RNA-Seq results are displayed as log_2_ Fold Change (ovary/testis), (*n* = 3 for each sex). qPCR results were evaluated by relative expression using *eef1b* and *rps4x* as reference genes and the optimized comparative Ct (2^-ΔΔCt^). (**C**) Consistency of log_2_ Fold Change(ovary/testis) between RNA-Seq data (*x*-axis) and qPCR data (*y*-axis) was high (R^2^ = 0.965) based on 20 genes.

In this study, 33,192 unigenes were identified as differentially expressed genes (DEGs) between the testes and ovaries (Figure 3A). Among these, 15,710 unigenes were up-regulated in the testis and 17,482 unigenes were up-regulated in the ovaries. Many of these DEGs were involved in sex determination and gonadal development, such as doublesex and mab-3 related transcription factor 1 (*dmrt1*), forkhead box protein L2 (*foxl2*), cytochrome P450s (*cyps*), izumo sperm-egg fusion protein 1 (*izumo1*), sperm flagellar proteins (*spefs*), wingless-type mice mammary tumor virus (MMTV) integration site family (*wnts*), zona pellucida sperm-binding proteins (*zps*), SRY (sex determining region Y)-box (*soxs*), and anti-Müllerian hormone (*amh*) (Table S6). The sequencing results for 22 PCR products (20 target genes and 2 references genes) indicated that the assembled unigenes were correct (data not shown). Further, we validated the gene expression patterns obtained by RNA-Seq using qPCR. The qPCR results were consistent with those of the RNA-Seq analysis (Figure 3B,C).

## 4. Discussion

### 4.1. SY-Medaka Belongs to O. curvinotus.

The morphological traits of fish are often used for species identification. The value 1.28 is a critical correlation coefficient for subspecies classification [34]. When the coefficient of variation of morphological traits between two populations exceeds this threshold, the populations can be considered subspecies; this measure only considers the morphological difference between geographical groups. SY-medaka and GQ-medaka have similar appearance (Figure S1). The variation of number of caudal fins was 0.47, the correlation coefficient of the length of pectoral fin was 0.58 (Table 1), both of them were less than the value of 1.28. Therefore, in terms of morphological characteristics, the differentiation between SY-medaka and GQ-medaka has not reached the subspecies level, and they can be considered different geographical groups of the same species.

Mitochondrial phylogenetic analyses of medaka indicate that the species can be divided into three major clades, referred to as the *latipes*, *javanicus*, and *celebensis* species groups (Figure 1B) [35,36]. Hellberg et al. (2009) proposed that when using mitochondrial genes as barcodes for the identification of animal species, the intraspecific genetic distance should not be more than 0.02, and the interspecific genetic distance should be significantly greater than the intraspecific distance [37]. In this study, the genetic distance between SY-medaka and *O. curvinotus* was only 0.005, much lower than the genetic distance between SY-medaka and *O. luzeonensis* of 0.095. Combined with morphological results and mitochondrial analysis, we determined that the SY-medaka population belongs to *O. curvinotus*.

### 4.2. SY-Medaka Does Not Contain dmy

The first sex-determining gene (*dmy*) was discovered in *O. latipes* [3], making it an ideal model for studies of sex determination and differentiation. Researchers have identified dozens of species in *Oryzias*, such as *O. latipes*, *O. sinensis*, *O. curvinotus*, *O. luzonensis*, *O. dancena*, *O. celebensis*, *O. mekongensis*, *O. javanicus*, and *O. melastigma* [38]. Previous studies have indicated that the sex chromosome type of *O. curvinotus* is XX/XY, and *dmy* is considered the sex-determining gene in this species [38]. However, SY-medaka may be an exception. In this study, nearly 300 individuals of SY-medaka were used for detection of *dmy*, and the results were negative (Figure 2A). Whole genome re-sequencing is currently the most comprehensive technology for determining the genetic basis of important traits [39]. In this study, whole genome re-sequencing reads for males and females of SY-medaka show low coverage of *dmy*, consistent with the re-sequencing results for female GQ-medaka (Figure 2B). In summary, based on molecular markers and whole genome re-sequencing, SY-medaka does not contain *dmy*. Previous studies have detected transcripts of *dmy* in the testis of mature *O. latipes* and *O. curvinotus* [3,5,6]. In this study, the nucleotide and protein sequences of *O. curvinotus dmy* were used for local BLAST searches, and *dmy* was not found, indicating that *dmy* expression is absent in SY-medaka.

### 4.3. Genes Related to Sex Determination and Gonadal Development in SY-Medaka

RNA-Seq analyses of gonads can be useful for the identification sex-related genes and candidate sex determination genes. We performed the first RNA-Seq analysis of SY-medaka gonads. The annotation rate (93.87% of 84,484 unigenes) was higher than those of the gonad transcriptomes of *S. sihama* (46.06% of 74,038 unigenes) [16] and *S. argus* (61.51% of 136,561). In a search against the NR database, 76.88% of annotated unigenes were found to be homologous to genes in *O. latipes* (Figure S4), reflecting the close evolutionary relationship between *O. curvinotus* and *O. latipes* [38]. Numerous DEGs related to male sex determination or testis development were identified by RNA-Seq, including *dmrt*1, *gsdf*, *sox*9, *amh*, *ars*, *smcs*, *hsd*17*b*, *hdacs*, and *izumo*1 (Table S6) [3,4,11,12,40,41,42,43,44]. In addition, several genes involved in female sex determination or ovarian development also were identified, such as *foxl*2, *cyp19a1a*, *cyps*, *zps*, *gdf9*, *zar*1, *bmps*, and *zglp*1 [15,45,46,47,48,49,50]. These findings provide a basis for research on sex determination, differentiation, and reproduction in *O. curvinotus*.

### 4.4. Lack of dmy in the SY-Medaka Genome

**Figure 4:**
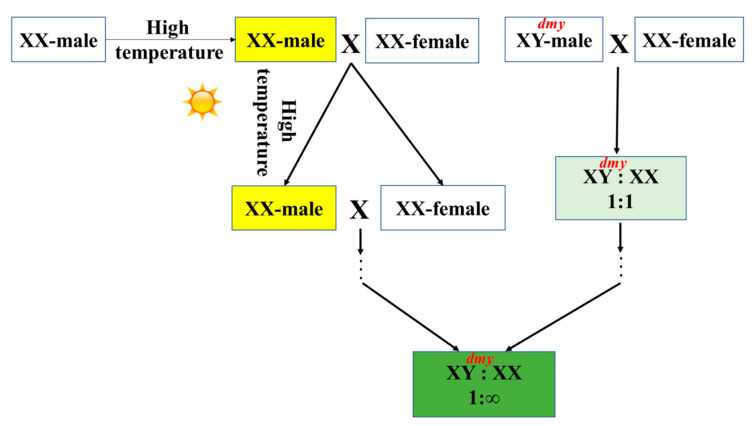
Schematic diagram of the evolution of *dmy* in SY-medaka (medaka in the Sanya River).

Fish sex determination is affected by genetic and environmental factors, and an important environmental factor is temperature [51]. Fish are sensitive to environmental conditions during gonad differentiation, and abnormal water temperatures can determine the direction of gonad differentiation [52,53]. High temperature often leads to the masculinization of fish [54,55,56,57]. Sanya is located in the tropics with high water temperatures, so we speculate that the SY-medaka lost *dmy* during evolution (Figure 4). Briefly, XX embryos of SY-medaka developed into males (XX-male) in high-temperature conditions; the XX-male, competitive with XY-male, mated with XX-female, which can only produce XX type offspring, increasing the ratio of XX in offspring. The ratio of XX in the population of SY-medaka accumulated over generations, which made the value of XX:XY became infinite, explaining the lack of XY individuals detected in our survey (Figure 4). Additional field surveys and reproduction experiments are needed to verify this hypothesis.

An alternative hypothesis is that SY-medaka has a sex determination gene other than *dmy*. Sex determination mechanisms vary among bony fishes, and species in the genus *Oryzias* harbor diverse sex-determining genes [38,51]. Three master sex-determining genes have been identified in *Oryzias* to date. In addition to *dmy*, which acts as a male sex-determining gene in *O. latipes* [3,4], *gsdfy* is a sex-determining gene in *O. luzonensis* [10], and sox3 serves as a sex-determining gene in O. dancena, O. *marmoratus*, and *O. profundicola* [30,57,58]. This diversity has inspired ongoing research on the molecular mechanisms underlying the rewiring of gene regulatory networks that are required to establish new master sex-determining genes [38,59,60].

## 5. Conclusions

In conclusion, we identified the SY-medaka population as *O curvinotus* based on morphological characteristics and a mitochondrial sequence analysis. Interestingly, sex-specific molecular markers and whole genome re-sequencing indicated that the SY-medaka genome lacks *dmy*. We also completed the first RNA-Seq analysis of the testis and ovary of SY-medaka and identified numerous DEGs related to sex determination and gonadal development. The RNA-Seq results also support the lack of *dmy* mRNA expression in SY-medaka. We speculate that the absence of *dmy* in SY-medaka can be explained by the high-temperature conditions, resulting in gene loss during evolution, or by the existence of another sex-determining gene. These findings provide a basis for further studies of the mechanism underlying sex determination in *Oryzias* and for studies focused on functional genomics and reproduction biology in *O. curvinotus*.

## Figures and Tables

**Table 1:** Comparison of morphological traits between GQ-medaka and SY-medaka.

Countable Traits	GQ-Medaka	SY-Medaka	Variable Coefficient
dorsal fin ray counts	6.02 ± 0.15	6.04 ± 0.19	0.05
anal fin ray counts	19.49 ± 0.87	19.71 ± 0.80	0.13
pectoral fin ray counts	8.09 ± 0.29	8.12 ± 0.32	0.05
caudal fin ray counts	18.51 ± 0.69 b	19.31 ± 1.02 a	0.47
ventral fin ray counts	6.00 ± 0.00	6.00 ± 0.00	
**measurable traits**			
Body weight/g	0.203 ± 0.06	0.204 ± 0.04	0.01
Total length/mm	27.71 ± 2.25 a	27.59 ± 1.83 b	0.03
Head length/mm	5.20 ± 0.62 b	5.44 ± 0.50 a	0.2
Snount length/mm	1.21 ± 0.25	1.29 ± 0.22	0.17
Eye orbit diameter/mm	2.06 ± 0.27	2.00 ± 0.22	0.12
Maximum depth of body/mm	5.01 ± 0.72	5.12 ± 0.44	0.09
Length of caudal fin/mm	4.32 ± 0.35	4.21 ± 0.31	0.17
Tips of snout to anus/mm	12.22 ± 0.94	12.04 ± 0.80	0.1
Tip of snout to dorsal fin/mm	13.14 ± 0.98 a	12.63 ± 0.92 b	0.27
Caudal peduncle length/mm	4.19 ± 0.33	4.17 ± 0.23	0.04
Caudal peduncle depth/mm	2.09 ± 0.25 b	2.24 ± 0.18 a	0.35
Length of dorsal fin/mm	3.30 ± 0.67 b	3.68 ± 0.67 a	0.28
Length of base of anal fin/mm	2.78 ± 0.35 b	2.96 ± 0.41 a	0.23
Length of pectoral fin/mm	3.90 ± 0.41 b	4.36 ± 0.37 a	0.58

Note: The value in the table is mean ± standard deviation (sd), different letters (a, b) indicate significant differences between GQ-medaka (Gaoqiao medaka) and SY-medaka (medaka in the Sanya River).

## Data Availability

All data generated or analyzed in this study are included in the submitted article and its supplementary information files. The raw RNA-Seq data were deposited at the CNGB Nucleotide Sequence Archive (CNSA) under the accession number CNP0000963 (https://db.cngb.org/search/?q=CNP0000963+).

## Supplementary Materials

The following are available online at https://www.mdpi.com/article/10.3390/ani11051327/s1, Figure S1: *O. curvinotus* form Gaoqiao and Sanya; Figure S2: Coverage of *dmrt*1 by whole genome re-sequencing reads of GQ-medaka and SY-medaka; Figure S3: Histological section of testis and ovary of SY-medaka; Figure S4: Distribution of homologous species of SY-medaka unigenes, Table S1: The sequences of primers used in this study; Table S2: The —sequencing results of 15 SY-medaka; Table S3: Estimates of evolutionary divergence between sequences; Table S4: Summary statistics of testes and ovaries transcriptome data of *O. curvinotus*; Table S5: *O. curvinotus* transcriptome reference assembly and annotation statistics; Table S6: The expression patterns of some DEGs related to gonad development and reproduction.
