# Supplementary material for: Oryzias curvinotus in Sanya Does Not Contain the Male Sex-Determining Gene dmy
Source: Animals (Basel). 2021 May 6;11(5):1327. doi: 10.3390/ani11051327 (PMC8148570; doi:10.3390/ani11051327)
Supplement: Supplementary file 1 [file animals-11-01327-s001.zip › animals-1196191-supplementary.pdf]

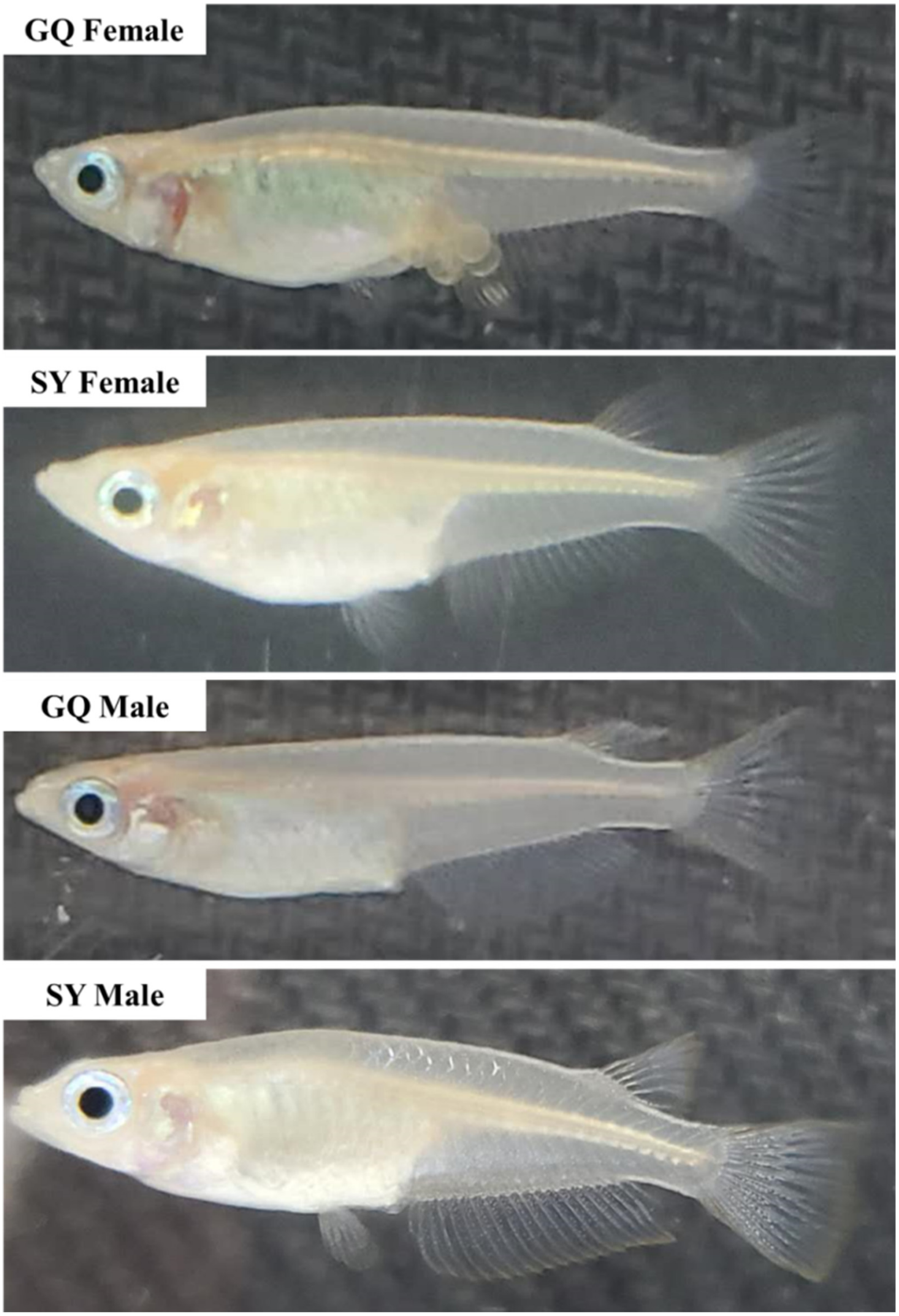

Figure S1. *O. curvinotus* form Gaoqiao and Sanya

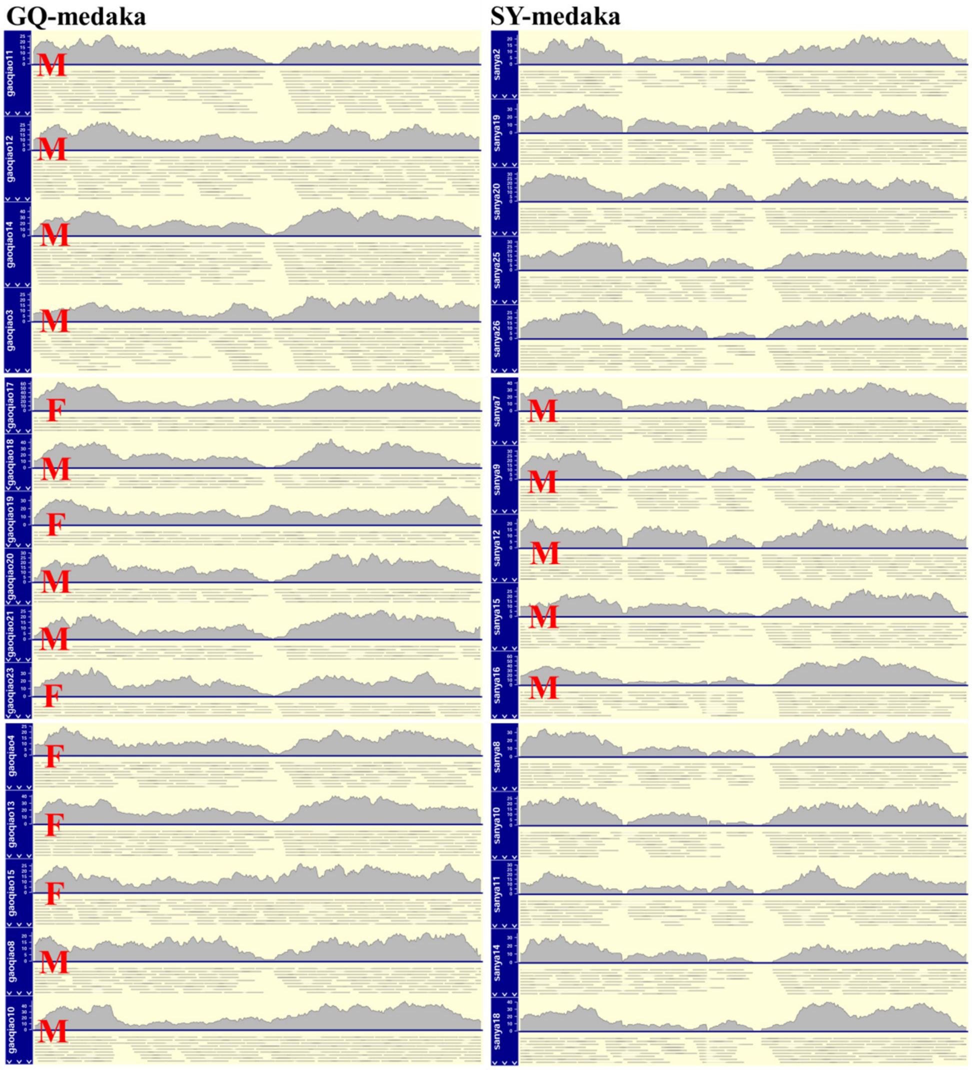

Figure S2. Coverage of *dmrt*1 by whole genome re-sequencing reads of GQ-medaka and SY-medaka. M indicates males and F indicates females.

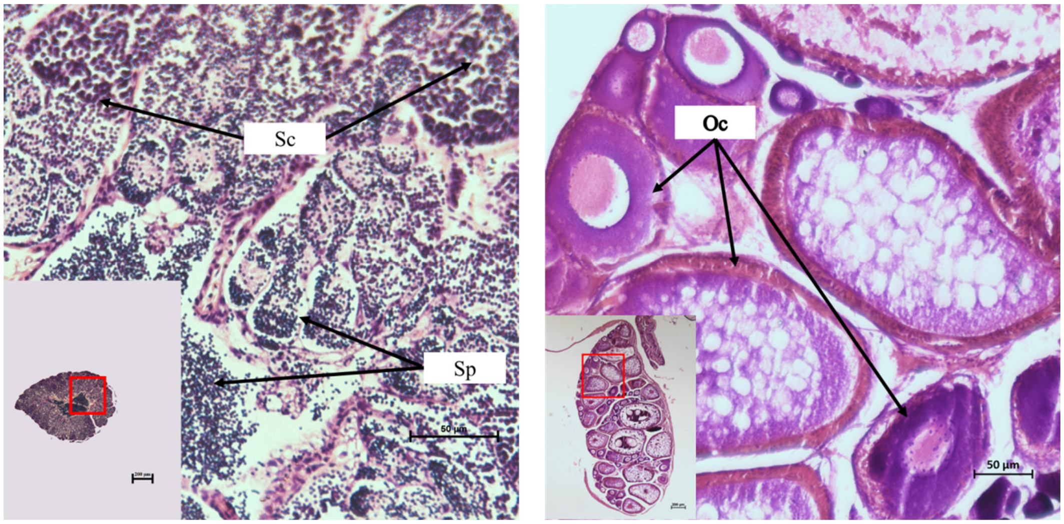

Figure S3. Histological section of testis and ovary of SY-medaka. SC, spermatocytes Sp, spermatids; OC, oocytes.

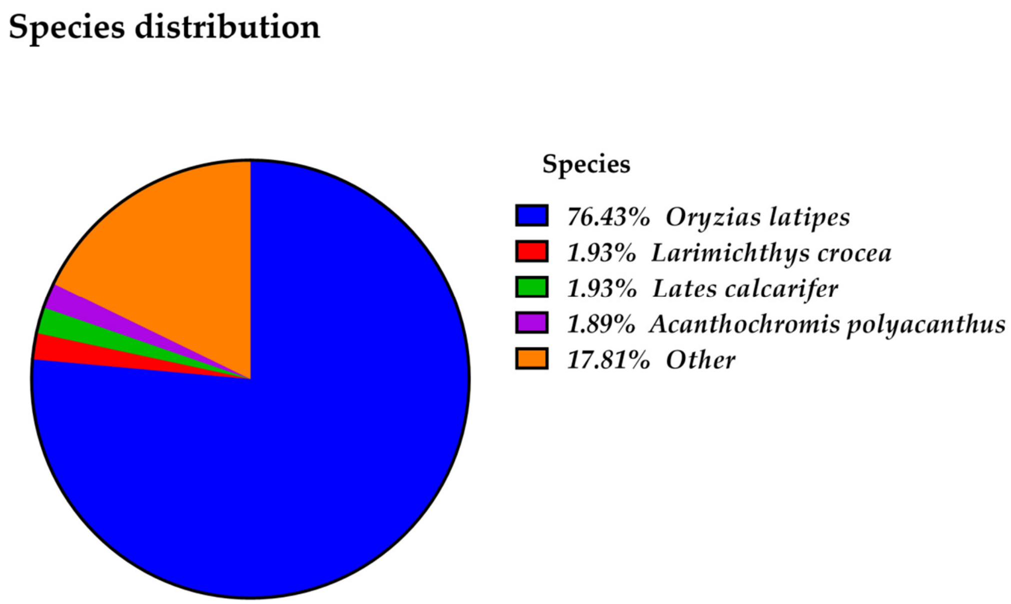

Figure S4. Distribution of homologous species of SY-medaka unigenes

Table S1. The sequences of primers used in this study

Primer name	Sequence 5′-3′	purpose
Co1-F	TCAACCAACCACAAAGACAATGGCAC	Species
Co1-R	TAGACTTCTGGGTGGCCAAAGAATCA	identification
Ocsex-F	ATGGTAACGCAGCCTTTCC	Genetic sex
Ocsex-R	GCCACATTCTTCTCAGGCA	determination
cbpa1	F: CTTCAACCACGAGCAATGTC, R: GGGAGTGTCCAGATTCAAAGTG	qPCR
odf3a	F: TCAGGGTGTAACTTGGTGCC, R: TCTTTGGCTGGGAGACTTCC	qPCR
cmbp4b	F: CGAAGACGCTACTCCTGATGTATC, R: CCTTTCTGTCCGCCTCAAAC	qPCR
saca4	F: TGTAACGAGGCACCTGAATCC, R: CACACACAGTCAAAGGCTTCTTC	qPCR
bmp15	F: CAAACTTCACTCCGTCACCC, R: GACAGTCTCCTTGGCAGTATCG	qPCR
h2a1a	F: TAGCACCACGCCACATCTTG, R: AGCAGCACCGTCTTCTTTCG	qPCR
zp4	F: TCTTGGAGGTCAACCCTGTTC, R: TCTACACAACCCTTTGCGAGAC	qPCR
horn	F: CATCAGAAACCTACAGGACAATCC, R: TCATCGTCGTCGTCACCATC	qPCR
loc101162755	F: GCTGAACCTTTGCCCTGATG, R: AGTGAGCGAGGAGGACCAAG	qPCR
dmrt1	F: CTTCATTCTTCACCACCACTTC, R: CGGAGCGTCAGAGAGTCAT	qPCR
zglp1	CCCAGAGAAGAAGCCAAGTTC, R: ATGGTGGTCAGCATCGTCAG	qPCR
loc101175599	F: TGTCAAACAAGAGAATGGGAGC, R: TCTGAAGTGGATGGAACTGTAGC	qPCR
spc1l	F: CATCAGGTCCATCAAGGCTTC, R: GAGGAGTGTGAAACCGTAGAGC	qPCR
nanos3	F: ACGGAGGTCAGTTTCGGATG, R: TAATGAACGGGTGAAGCGTG	qPCR
gsdf	F: TAGCCTATCTTGACGGACAGCC, R: GCTCTTCCCTTGATGGATGC	qPCR
insl5	F: GTCAGCCCGTCAACCAGAATC, R: CAAACATCTCACAGGAAAGGAGG	qPCR
cyp19a	F: CTCTTCCTGGGTGTTCCTGTTG, R: TCTTGTGCCTCTGATGAATCC	qPCR
izumo1	F: GGTCTCAGCAGCAGGGATAAAG, R: AGGAAATCATCAGAACCCAGAGTG	qPCR
mlf1	F: GCTTCAGACTCATCCAGGTTC, R: GCCACTACATCCAAGACAGAGG	qPCR
foxl2	F: TTCAAGACAGAAGCAGCAGTATTC, R: GCCGATGACACCTTTATCTCC	qPCR
ee1b	F:GATGATGACATTGACCTGTTTGG, R:GATGGAGGACTTGGCGATG	qPCR
rps4x	F:CGAAACACTGGATGCTGGATAAG, R:ACTTCAGGCGGTTCCTCAGG	qPCR

Table S2. The COI sequencing results of 15 SY-medaka

Name	Sequence 5`-3`
COI_1#	AAAAATGTTGTATTTAGGTTCCGATCTGTGAGAAGCATGGTGATACCTGCAGCTAGAACAGGAAGAGA<br>GAGTAGAAGTAATACTGCGGTAATTAGTACAGCCCACACAAATAAAGGGGTTTGATATTGGGAAATGG<br>CTGGAGGTTTTATATTAATAATAGTTGTGATGAAATTAATGGCCCCTAGGATAGAAGAAATTCCGGCCAG<br>GTGAAGAGAGAAAATGGTTAAATCTACGGAGGCCCCTGCGTGTGCCAAATTACCTGACAAGGGGGGA<br>TATACGGTTCATCCTGTTCCGGCACCAGCTTCTACACCAGATGAGGCCAATAATAGAAGGAAAGAAGG<br>GGGCAGGAGTCAAAAGCTTATATTATTTATCCGGGGGAAGGCCATGTCGGGGGCTCCAATCATTAAAG<br>GGATTAATCAGTTGCCAAACCCCCCAATTATGATTGGCATTACTATAAAGAAAATTATTACGAAAGCAT<br>GTGCAGTTACAATTACGTTATAGATCTGGTCGTCTCCTAATAGGGAGCCTGGTTGACTTAGTTCTGCCCG<br>AATGAGTAGACTTAAGGCCGTCCCTACCATTCCCGCCCAGGCACCAAAGATTAGATACAGGGTGCCAT<br>TGTCTTTGTGGTT
COI_2#	TCCGATCTGTGAGAAGCATGGTGATACCTGCAGCTAGAACAGGAAGAGAGAGTAGAAGTAATACTGC<br>GGTAATTAGTACAGCCCACACAAATAAAGGGGTTTGATATTGGGAAATGGCTGGAGGTTTTATATTAAT<br>AATAGTTGTGATGAAATTAATGGCCCCTAGGATAGAAGAAATTCCGGCCAGGTGAAGAGAGAAAATGG<br>TTAAATCTACGGAGGCCCCTGCGTGTGCCAAATTACCTGACAAGGGGGGATATACGGTTCATCCTGTTC<br>CGGCACCAGCTTCTACACCAGATGAGGCCAATAATAGAAGGAAAGAAGGGGGCAGGAGTCAAAAGC<br>TTATATTATTTATCCGGGGGAAGGCCATGTCGGGGGCTCCAATCATTAAAGGGATTAATCAGTTGCCAA<br>ACCCCCCAATTATGATTGGCATTACTATAAAGAAAATTATTACGAAAGCATGTGCAGTTACAATTACGTT<br>ATAGATCTGGTCGTCTCCTAATAGGGAGCCTGGTTGACTTAGTTCTGCCCGAATGAGTAGACTTAAGGC<br>CGTCCCTACCATT
COI_3#	TTCCGATCTGTGAGAAGCATGGTGATACCTGCAGCTAGAACAGGAAGAGAGAGTAGAAGTAATACTGC<br>GGTAATTAGTACAGCCCACACAAATAAAGGGGTTTGATATTGGGAAATGGCTGGAGGTTTTATATTAAT<br>AATAGTTGTGATGAAATTAATGGCCCCTAGGATAGAAGAAATTCCGGCCAGGTGAAGAGAGAAAATGG<br>TTAAATCTACGGAGGCCCCTGCGTGTGCCAAATTACCTGACAAGGGGGGATATACGGTTCATCCTGTTC<br>CGGCACCAGCTTCTACACCAGATGAGGCCAATAATAGAAGGAAAGAAGGGGGCAGGAGTCAAAAGC<br>TTATATTATTTATCCGGGGGAAGGCCATGTCGGGGGCTCCAATCATTAAAGGGATTAATCAGTTGCCAA<br>ACCCCCCAATTATGATTGGCATTACTATAAAGAAAATTATTACGAAAGCATGTGCAGTTACAATTACGTT<br>ATAGATCTGGTCGTCTCCTAATAGGGAGCCTGGTTGACTTAGTTCTGCCCGAATGAGTAGACTTAAGGC<br>CGTCCCTACCATTCCCGCCCAGGCACCAAAGATTAGATACAGGGTGCCATTGTCTT
COI_4#	AAGAGAGAGTAAAAGTAATACTGCGGTAATTAGTACAGCCCACACAAATAAAGGGGTTTGATATTGGG<br>AAATGGCTGGAGGTTTTATATTAATAATAGTTGTGATGAAATTAATGGCCCCCAGGATAGAAGAAATTC<br>CGGCCAGGTGAAGAGAGAAAATGGTTAAATCTACGGAGGCCCCTGCGTGTGCCAAATTACCTGACAA<br>GGGGGGATATACGGTTCATCCTGTTCCGGCACCAGCTTCTACACCAGATGAGGCCAATAATAGAAGGA<br>AAGAAGGGGGCAGGAGTCAAAAGCTTATATTATTTATCCGGGGGAAGGCCATGTCGGGGGCTCCAATC<br>ATTAAAGGGATTAATCAGTTGCCAAACCCCCCAATTATGATTGGCATTACTATAAAGAAAATTATTACGA<br>AAGCATGTGCAGTTACAATTACGTTATAGATCTGGTCGTCTCCTAATAGGGAGCCTGGTTGACTTAGTTC<br>TGCCCGAATGAGTAGACTTAAGGCCGTCCCTACCATTCCCG
COI_5#	AACAGGAAGAGAGAGTAGAAGTAATACTGCGGTAATTAGTACAGCTCACACAAATAAAGGGGTTTGAT<br>ATTGGGAAATGGCTGGAGGTTTTATATTAATAATAGTTGTGATGAAATTAATGGCCCCTAGGATAGAAGA<br>AATTCCGGCCAGGTGAAGAGAGAAAATGGTTAAATCTACGGAGGCCCCTGCGTGTGCCAAATTACCTG<br>ACAAGGGGGGATATACGGTTCATCCTGTCCCGGCACCAGCTTCTACACCAGATGAGGCCAATAATAGA<br>AGGAAAGAAGGGGGCAGGAGTCAAAAGCTTATATTATTTATCCGTGGGAAGGCCATGTCGGGGGCTCC<br>AATCATTAAAGGAATTAATCAGTTGCCAAACCCCCCAATTATGATTGGCATTACTATAAAGAAAATTATT<br>ACGAAAGCATGTGCAGTTACAATTACGTTATAGATCTGGTCGTCTCCTAATAGGGAGCCTGGTTGACTT<br>AGTTCTGCCCGAATGAGTAGACTTAAGGCCGTCCCCACCATTCCCGCCCAGGCACCAAAGATTAGATA<br>CAGGGTGCCATTGTCTTTGTGGTTTGG
COI_6#	TGAGAAGCATGGTGATACCTGCAGCTAGAACAGGAAGAGAGAGTAGAAGTAATACTGCGGTAATTAGT<br>ACAGCCCACACAAATAAAGGGGTTTGATATTGGGAAATGGCTGGAGGTTTTATATTAATAATAGTTGTG<br>ATGAAATTAATGGCCCCTAGGATAGAAGAAATTCCGGCCAGGTGAAGAGAGAAAATGGTTAAATCTAC<br>GGAGGCCCCTGCGTGTGCCAAATTACCTGACAAGGGGGGATATACGGTTCATCCTGTTCCGGCACCAG<br>CTTCTACACCAGATGAGGCCAATAATAGAAGGAAAGAAGGGGGCAGGAGTCAAAAGCTTATATTATTT<br>ATCCGGGGGAAGGCCATGTCGGGGGCTCCAATCATTAAAGGGATTAATCAGTTGCCAAACCCCCCAAT<br>TATGATTGGCATTACTATAAAGAAAATTATTACGAAAGCATGTGCAGTTACAATTACGTTATAGATCTGG<br>TCGTCTCCTAATAGGGAGCCTGGTTGACTTAGTTCTGCCCGAATGAGTAGACTTAAGGCCGTCCCTACC<br>ATTCCCGCCCAGGCACCAAAGATTAGATACAGG
COI_7#	AGGAAGAGAGAGTAGAAGTAATACTGCGGTAATTAGTACAGCCCACACAAATAAGGGGGTTTGATATT

	GGGAAATGGCTGGAGGTTTTATATTAATAATAGTTGTGATGAAATTAATGGCCCCCAGGATAGAAGAAA<br>TTCCGGCCAGGTGAAGAGAGAAAATGGTTAAATCTACGGAGGCCCCTGCGTGTGCCAAATTACCTGAC<br>AAGGGGGGATATACGGTTCATCCTGTTCCGGCACCAGCTTCTACACCAGATGAGGCCAATAATAGAAG<br>GAAAGAAGGGGGCAGGAGTCAAAAGCTTATATTATTTATCCGGGGGAAGGCCATGTCGGGGGCTCCAA<br>TCATTAAAGGGATTAATCAGTTGCCAAACCCCCCAATTATGATTGGCATTACTATAAAGAAAATTATTAC<br>GAAAGCATGTGCAGTTACAATTACGTTATAGATCTGGTCGTCTCCTAATAGGGAGCCTGGTTGACTTAGT<br>TCTGCCCGAATGAGTAGACTTAAGGCCGTCCCTACCATTCCCGCCCAGGCACCAAAGA
COI_8#	TAGGTTCCGATCTGTGAGAAGCATGGTGATACCTGCAGCTAGAACAGGAAGAGAGAGTAGAAGTAATA<br>CTGCGGTAATTAGTACAGCCCACACAAATAAAGGGGTTTGATATTGGGAAATGGCTGGAGGTTTTATAT<br>TAATAATAGTTGTGATGAAATTAATGGCCCCTAGGATAGAAGAAATTCCGGCCAGGTGAAGAGAGAAA<br>ATGGTTAAATCTACGGAGGCCCCTGCGTGTGCCAAATTACCTGACAAGGGGGGATATACGGTTCATCCT<br>GTTCCGGCACCAGCTTCTACACCAGATGAGGCCAATAATAGAAGGAAAGAAGGGGGCAGGAGTCAAA<br>AGCTTATATTATTTATCCGGGGGAAGGCCATGTCGGGGGCTCCAATCATTAAAGGGATTAATCAGTTGCC<br>AAACCCCCCAATTATGATTGGCATTACTATAAAGAAAATTATTACGAAAGCATGTGCAGTTACAATTAC<br>GTTATAGATCTGGTCGTCTCCTAATAGGGAGCCTGGTTGACTTAGTTCTGCCCGAATGAGTAGACTTAAG<br>GCCGTCCCTACCATTCCCGCCCAGGCACCAAA
COI_9#	GGTCGAAAAATGTTGTATTTAGGTTCCGATCTGTGAGAAGCATGGTGATACCTGCAGCTAGAACAGGA<br>AGAGAGAGTAGAAGTAATACTGCGGTAATTAGTACAGCCCACACAAATAAAGGGGTTTGATATTGGGA<br>AATGGCTGGAGGTTTTATATTAATAATAGTTGTGATGAAATTAATGGCCCCTAGGATAGAAGAAATTCCG<br>GCCAGGTGAAGAGAGAAAATGGTTAAATCTACGGAGGCCCCTGCGTGTGCCAAATTACCTGACAAGG<br>GGGGATATACGGTTCATCCTGTTCCGGCACCAGCTTCTACACCAGATGAGGCCAATAATAGAAGGAAA<br>GAAGGGGGCAGGAGTCAAAAGCTTATATTATTTATCCGGGGGAAGGCCATGTCGGGGGCTCCAATCAT<br>TAAAGGGATTAATCAGTTGCCAAACCCCCCAATTATGATTGGCATTACTATAAAGAAAATTATTACGAA<br>AGCATGTGCAGTTACAATTACGTTATAGATCTGGTCGTCTCCTAATAGGGAGCCTGGTTGACTTAGTTCT<br>GCCCGAATGAGTAGACTTAAGGCCGTCCCTACCATTCCCGCCCAGGCACCAAAGATTAGATACAGGGT<br>GCCATTGTC
COI_10#	GGTCGAAAAATGTTGTATTTAGGTTCCGATCTGTGAGAAGCATGGTGATACCTGCAGCTAGAACAGGA<br>AGAGAGAGTAGAAGTAATACTGCGGTAATTAGTACAGCCCACACAAATAAAGGGGTTTGATATTGGGA<br>AATGGCTGGAGGTTTTATGTTAATAATAGTTGTGATGAAATTAATGGCCCCTAGGATGGAAGAAATTCC<br>GGCCAGGTGAAGAGAGAAAATGGTTAAATCTACGGAGGCCCCTGCGTGTGCCAAATTACCTGACAAG<br>GGGGGATATACGGTTCATCCTGTTCCGGCACCAGCTTCTACACCAGATGAGGCCAATAATAGAAGGAA<br>AGAAGGGGGCAGGAGTCAAAAGCTTATATTATTTATCCGTGGGAAGGCCATGTCGGGGGCTCCAATCA<br>TTAAAGGGATTAATCAGTTGCCAAACCCCCCAATTATGATTGGCATTACTATAAAGAAAATTATTACGA<br>AAGCATGTGCAGTTACAATTACGTTATAGATCTGGTCGTCTCCTAATAGGGAGCCTGGTTGACTTAGTTC<br>TGCCCGAATGAGTAGACTTAAGGCCGTCCCTACCATTCCCGCCCAGGCACCAAAGATTAGATACAGGG<br>TG
COI_11#	CGAAAAATGTTGTATTTAGGTTTCGATCTGTGAGAAGCATGGTGATACCTGCAGCTAGAACAGGAAGG<br>GAGAGTAGAAGTAATACTGCGGTAATTAGTACAGCTCACACAAATAAAGGGGTTTGATATTGGGAAAT<br>GGCTGGGGGTTTTATGTTAATAATAGTTGTGATGAAATTAATGGCCCCTAGGATAGAAGAAATTCCGGC<br>CAGGTGAAGAGAGAAAATGGTTAAATCTACGGAGGCCCCTGCGTGTGCCAAATTACCTGACAAGGGG<br>GGATATACGGTTCATCCTGTTCCGGCACCAGCTTCTACACCAGATGAGGCCAATAAAAGAAGGAAAGA<br>AGGGGGCAGGAGTCAAAAGCTTATATTATTTATCCGTGGGAAGGCCATGTCGGGGGCTCCAATCATTAA<br>AGGAATTAATCAGTTGCCAAACCCCCCAATTATGATTGGCATTACTATAAAGAAAATTATTACGAAAGC<br>ATGTGCAGTTACAATTACGTTATAGATCTGGTCGTCTCCTAATAGGGAGCCTGGTTGACTTAGTTCTGCC<br>CGAATGAGTAGACTTAAGGCCGTCCCTACCATTCCCGCCCAGGCACCAAAGA
COI_12#	GGGGTCGAAAAATGTTGTATTTAGGTTTCGATCTGTGAGAAGCATAGTGATACCTGCAGCTAGAACAGG<br>AAGAGAGAGTAGGAGTAATACTGCGGTAATTAGAACAGCTCACACAAATAAAGGGGTTTGATATTGGG<br>AGATGGCTGGAGGTTTTATGTTAATAATAGTTGTAATGAAATTAATGGCCCCTAGAATAGAAGAAATTCC<br>GGCCAGGTGAAGAGAGAAAATGGTTAAATCTACGGAGGCCCCTGCGTGTGCCAAATTACCGGACAAG<br>GGGGGATATACGGTTCATCCTGTCCCGGCACCAGCTTCTACACCAGATGAGGCCAATAAAAGAAGGAA<br>AGAAGGGGGCAGGAGTCAAAAGCTTATATTATTTATCCGGGGGAAGGCCATGTCGGGAGCTCCGATCA<br>TTAAAGGAATTAATCAGTTGCCGAACCCTCCAATTATAATTGGCATTACTATAAAGAAAATTATTACGAA<br>AGCATGTGCAGTTACAATTACGTTATAGATCTGGTCGTCTCCTAATAGGGAGCCTGGTTGACTTAATTCT<br>GCCCGAATGAGCAGACTTAAGGCCGTCCCTACTATTCCCGCCCAGGCACCAAA
COI_13#	GTATTTAGGTTCCGATCTGTGAGAAGTATGGTGATTCCTGCAGCTAGAACAGGAAGAGAGAGTAGAAG<br>TAATACTGCGGTAATTAGTACAGCCCACACAAATAAGGGGGTTTGATATTGAGAAATGGCTGGAGGTTT<br>CATGTTAATAATTGTTGTGATGAAATTAATGGCCCCTAAGATGGAAGAAATTCCTGCCAGGTGAAGAGA

	GAAAATGGTTAAGTCTACGGATGCCCCTGCGTGTGCCAAATTACCTGACAAGGGGGGATATACGGTTC<br>ATCCTGTTCCGGCACCAGCTTCTACACCAGATGAAGCCAATAAAAGAAGGAAAGAAGGGGGCAGGAG<br>TCAAAAGCTTATATTATTTATCCGAGGGAAGGCCATGTCTGGGGCTCCAATCATTAAAGGGATTAATCA<br>GTTGCCAAACCCCCCAATTATGATTGGCATTACTATAAAGAAAATTATTACGAAAGCATGTGCAGTTAC<br>AATTACGTTATAAATCTGGTCGTCTCCTAATAGAGAGCCTGGTTGACTTAATTCTGCCCGA
COI_14#	TGTATTTAGGTTCCGATCTGTGAGAAGTTGGTGATTCCTGCAGCTAGAACAGGAAGAGAGAGTAGAAG<br>TAATACTGCGGTAATTAGTACAGCCCACACAAATAAAGGGGTTTGATATTGGGAAATGGCTGGAGGTTT<br>CATGTTAATAATTGTTGTGATGAAATTAATGGCCCCTAAGATGGAAGAAATTCCTGCCAGGTGAAGAGA<br>GAAAATGGTTAAGTCTACGGATGCCCCTGCGTGTGCCAAATTACCTGACAAGGGGGGATATACGGTTC<br>ATCCTGTTCCGGCACCAGCTTCTACACCAGATGAGGCCAATAAAAGAAGGAAAGAAGGGGGCAGGAG<br>TCAAAAGCTTATATTATTTATCCGGGGGAAGGCCATGTCTGGGGCTCCAATCATTAAAGGGATTAATCA<br>GTTGCCAAACCCCCCAATTATGATTGGCATTACTATAAAGAAAATTATTACGAAAGCATGTGCAGTTAC<br>AATTACGTTATAAATCTGGTCGTCTCCTAATAGAGAGCCTGGTTGACTTAATTCTGCCCG
COI_15#	CCTGCGGGGTCGAAGATGTTGTATTTAGGTTCCGATCTGTGAGAAGTATGGTGATTCCTGCAGCTAGAA<br>CAGGAAGGGAGAGTAGAAGTAAAACTGCGGTAATTAGTACAGCTCACACAAATAAGGGGGTTTGATA<br>TTGGGAAATGGCTGGGGGTTTCATGTTAATAATTGTTGTGATGAAATTAATGGCCCCTAAGATGGAAGA<br>AATTCCTGCCAGGTGAAGAGAGAAAATGGTTAAGTCTACGGATGCCCCTGCGTGTGCCAAATTCCCTG<br>ACAAGGGAGGATATACGGTTCATCCTGTTCCGGCACCAGCTTCTACACCAGATGAAGCCAATAAAAGA<br>AGGAAAGAAGGGGGCAGGAGTCAAAAGCTTATATTATTTATCCGAGGGAAGGCCATGTCTGGGGCTCC<br>AATCATTAAAGGAATTAATCAGTTGCCAAACCCCCCAATTATGATTGGCATTACTATAAAGAAAATTATT<br>ACGAAAGCATGTGCAGTTACAATTACGTTATAAATCTGGTCGTCTC

Table S3. Estimates of Evolutionary Divergence between Sequences

	SY-medaka	*Ocu*	*Olu*	*Ola*	*Osi*	*Oja*	*Omi*	*Oda*	*Ome*	*Oce*	*Osa*	*Oma*	*Dre*
SY-medaka													
Ocu	0.005												
Olu	0.095	0.094											
Ola	0.135	0.135	0.144										
Osi	0.137	0.137	0.145	0.107									
Oja	0.184	0.185	0.191	0.186	0.188								
Omi	0.204	0.204	0.214	0.205	0.206	0.187							
Oda	0.187	0.186	0.200	0.191	0.187	0.169	0.181						
Ome	0.185	0.185	0.200	0.189	0.187	0.169	0.180	0.010					
Oce	0.189	0.189	0.199	0.192	0.188	0.195	0.199	0.184	0.184				
Osa	0.192	0.191	0.198	0.194	0.189	0.199	0.208	0.193	0.193	0.096			
Oma	0.188	0.187	0.196	0.191	0.188	0.198	0.204	0.190	0.189	0.093	0.070		
Dre	0.266	0.265	0.272	0.272	0.267	0.275	0.272	0.267	0.267	0.263	0.266	0.263	

Ocu, O. curvinotus; *Olu, O. luzonensis*; *Ola, O. latipes*; *Osi, O. sinensis*; *Oja, O. javanicus*; *Omi, O. minutillus*; *Oda, O. dancena*; *Ome, O. melastigma*; *Oce, O. Celebensis*; *Osa, O. sarasinorum*; *Oma, O. marmoratus*; *Dre, D. rerio.* The mitochondrial sequences ID of those fish are shown in Figure. 1B.

Table S4. Summary statistics of testes and ovaries transcriptome data of *O. curvinotus*

Sample	ovary_SY1	ovary_SY2	ovary_SY3	testis_SY1	testis_SY2	testis_SY3
Raw Reads(M)	63.23	62.86	63.15	62.85	63.53	63.27
Clean Reads(M)	61.01	60.92	61.14	61.2	61.59	61.08
Clean Reads Q20(%)	98.35	98.53	98.38	98.57	98.68	98.39
Clean Reads Q30(%)	94.25	95.06	94.21	95.03	95.41	94.25
GC content (%)	49.23	49.03	48.89	49.16	48.32	49.01
Transcripts number	64,959	66,276	72,248	75,928	98,055	87,829
Unigenes number	49,135	49,232	52,952	55,270	61,902	60,810

Table S5. *O. curvinotus* transcriptome reference assembly and annotation statistics

Database	Number	Percentage (%)
**Assembly**		
Total base (bp)	94,333,707	
Number of unigenes	84,484	
Average length (bp)	1,116	
N50 (bp)	2,384	
GC content		48.67
**Annotation**		
Annotated in NR	46,377	54.89
Annotated in NT	78,492	92.91
Annotated in KEGG	37,636	44.55
Annotated in SwissProt	37,843	44.79
Annotated in KOG	33,324	39.44
Annotated in GO	10,221	12.10
Overall	79,298	93.87

Table S6. The expression patterns of some DEGs related to gonad development and reproduction

Gene	Gene annotation	Probabilit	Nr ID	log2FC(ovary/testi
wnt7b	wingless-type MMTV integration site family, member 7B	1	XP_020569989.	-7.39518
wnt4b	wingless-type MMTV integration site family member 4b	1	NP_001153912.	-8.82761
wnt2	wingless-type MMTV integration site family member 2	0.999605	XP_020492875.	-7.49185
trd7b	Tudor domain-containing protein 7B	0.999051	XP_004079778.	-2.67008
theg	Testicular haploid expressed gene protein	0.999995	XP_020569141.	-6.79008
tdr15	Tudor domain-containing protein 15	1	XP_018550215.	-3.03149
star9	StAR-related lipid transfer protein 9	0.998506	XP_020569428.	-2.44163
spef2	Sperm flagellar protein 2	1	XP_020561437.	-8.77329
spef1	Sperm flagellar protein 1	1	XP_004077227.	-5.21739
spc1l	Speriolin-like protein	1	XP_020565798.	-14.1918
spat7	Spermatogenesis-associated protein 7	0.999657	XP_011488521.	-2.11963
sox9	SRY (sex determining region Y)-box 9	1	BAH05019.1	-4.37676
smc1b	Structural maintenance of chromosomes protein 1B	1	XP_017285706.	-8.81513
smc3	structural maintenance of chromosomes protein 3	0.99944643	XP_004066378.	-2.00674226
rec8	Meiotic recombination protein REC8 homolog	1	XP_004081264.	-5.22288
r3hd4	R3H domain-containing protein 4	0.999294	XP_004068025.	-7.89785
pol5	Retrovirus-related Pol polyprotein from transposon opus	0.999049	XP_008293872.	-3.43296
pol4	Retrovirus-related Pol polyprotein from transposon 412	0.995518	XP_019221790.	-3.55639
pol3	Retrovirus-related Pol polyprotein from transposon 17.6	0.993254	XP_008283279.	-2.49442
pk3cb	Phosphatidylinositol 4,5-bisphosphate 3-kinase catalytic subunit beta	0.982855	XP_011481352.	-2.70834
mlf1	Myeloid leukemia factor 1	1	XP_011481028.	-8.19251
kat7	Histone acetyltransferase KAT7	1	XP_020561190.	-5.26325
kat5	Histone acetyltransferase KAT5	0.998634	XP_011481726.	-3.41885
kapca	cAMP-dependent protein kinase catalytic subunit alpha	0.999524	XP_008434985.	-5.11088
izumo1	Izumo sperm-egg fusion protein 1	1	XP_020561433.	-8.92296
itpr3	Inositol 1,4,5-trisphosphate receptor type 3	0.998897	XP_020557263.	-4.1838
itpr1	Inositol 1,4,5-trisphosphate receptor type 1	0.986726	XP_020559132.	-2.36535
hsp70	Heat shock cognate 70 kDa protein	0.999753	NP_001098385.	-7.06286
hsd17b	hydroxysteroid 17-beta dehydrogenase 7	0.998642	XP_017280664.	-4.72247
hs90a	Heat shock protein HSP 90-alpha	0.998449	XP_004083819.	-2.81199
hdac8	Histone deacetylase 8	0.999995	XP_011485972.	-2.53244
hdac7	Histone deacetylase 7	0.999047	XP_011473874.	-2.90754
hdac3	Histone deacetylase 3	1	XP_004073599.	-2.75168
gtd2b	General transcription factor II-I repeat domain-containing protein 2B	1	XP_004077286.	-9.38298
gsdf	gonadal soma derived factor	0.999904	NP_001171213.	-2.92127
gphb5	Glycoprotein hormone beta-5	0.998848	XP_020567290.	-7.88874
gnrr2	gonadotropin-releasing hormone receptor 2	1	NP_001098392.	-9.34725
gnaq	Guanine nucleotide-binding protein G(q) subunit alpha	0.99888	XP_020556761.	-2.18813
gna14	Guanine nucleotide-binding protein subunit alpha-14	0.998538	XP_004074607.	-2.21025
gin1	Gypsy retrotransposon integrase-like protein 1	0.996027	XP_016415952.	-4.12928
gata5	Transcription factor GATA-5	0.998401	XP_011475114.	-3.54386
fgfr3	Fibroblast growth factor receptor 3	0.995207	XP_004082745.	-2.50714
dmrt1	Doublesex- and mab-3-related transcription factor 1	1	AAS91465.1	-9.7535
dhcr7	7-dehydrocholesterol reductase	0.998121	XP_022071062.	-2.6881
dhc24	Delta(24)-sterol reductase	1	XP_018525864.	-8.27922
dh12b	Very-long-chain 3-oxoacyl-CoA reductase-B	0.982641	XP_011485867.	-2.36457
cpeb4	Cytoplasmic polyadenylation element-binding protein 4	0.999635	XP_011481506.	-4.70769
cp4b1	cytochrome P450 4B1-like	0.999993	XP_004078318.	-6.35814
cp26b	cytochrome P450, family 26, subfamily B	0.998745	NP_001265750.	-5.564
cp11b	cytochrome P450 11beta	1	NP_001098570.	-7.91754
cfa70	Cilia- and flagella-associated protein 70	1	XP_020567678.	-8.26835
cfa54	Cilia- and flagella-associated protein 54	0.99999	XP_020564512.	-6.77196
cb5d1	cytochrome b5 domain-containing protein 1	1	XP_004086762.	-7.56122
amh	anti-Mullerian hormone	0.984112	NP_001098198.	-1.0285
akt3	RAC-gamma serine/threonine-protein kinase	0.999764	XP_007570539.	-3.84082
zp4	ona pellucida sperm-binding protein 4	1	XP_011472723.	4.20555243
zp3	ona pellucida sperm-binding protein 3	1	XP_011488823.	12.42472542
zp1	ona pellucida sperm-binding protein 1	1	XP_004071858.	4.428162622
zglp1	zinc finger, GATA-like protein 1	1	XP_004065851.	7.585234067

zar1	Zygote arrest protein 1	1	XP_004067611.	9.217390254
wnt9b	wingless-type MMTV integration site family, member 9B	0.99999958	XP_020560833.	7.686500527
wnt9a	wingless-type MMTV integration site family member 9a	0.99698495	XP_004079455.	4.041963293
wnt5b	wingless-type MMTV integration site family, member 5B	0.99752726	XP_011489302.	3.348832279
wnt5a	wingless-type MMTV integration site family, member 5A	0.99941565	XP_022059770.	4.371022644
wnt4a	wingless-type MMTV integration site family member 4a	0.99840374	NP_001153911.	2.032069901
wnt16	wingless-type MMTV integration site family, member 16	0.99881709	XP_020557844.	5.32309109
wn2bb	wingless-type MMTV integration site family, member 2Bb	0.99158103	XP_011475046.	2.704871964
wif1	Wnt inhibitory factor 1	0.99970288	XP_020558359.	5.764871591
tf7l2	Transcription factor 7-like 2	0.99546189	XP_022046618.	2.036951136
tesk1	Dual specificity testis-specific protein kinase 1	0.99980362	XP_011487062.	2.161067814
sx17a	SRY (sex determining region Y)-box 17	0.99785707	NP_001158345.	2.515945327
strbp	Spermatid perinuclear RNA-binding protein	0.99693893	XP_018525986.	2.205448658
spat1	Spermatogenesis-associated protein 1	0.99378780	XP_011472245.	2.683194338
sp130	Histone deacetylase complex subunit SAP130	1	XP_011472693.	3.005244672
sox7	SRY (sex determining region Y)-box 7	0.99889622	XP_011490013.	2.484079144
sox10	SRY (sex determining region Y)-box 10	0.99951725	NP_001158343.	5.294620749
sox6	SRY (sex determining region Y)-box 6	0.94845405	XP_019213816.	1.131911676
sox17	SRY (sex determining region Y)-box 17	0.96333333	NP_001158345.	2.515945327
sx18a	SRY (sex determining region Y)-box 18	0.92435533	XP_011475950.	1.071622928
sox5	SRY (sex determining region Y)-box 5	0.98125103	BAO57768.1	1.413397141
sox4	SRY (sex determining region Y)-box 4	0.99488196	XP_020555578.	1.876193798
rspo3	R-spondin-3	0.99966952	XP_015250864.	6.942514505
rarga	Retinoic acid receptor gamma-A	0.99748218	XP_004069175.	4.611024797
pthd1	Patched domain-containing protein 1	0.99858132	XP_011487428.	4.442943496
ptc1	Protein patched homolog 1	0.99540125	XP_011485123.	2.329214449
nr6a1	Nuclear receptor subfamily 6 group A member 1-A	0.99977779	XP_007557810.	4.218834602
nr5a2	Nuclear receptor subfamily 5 group A member 2	1	NP_001098298.	3.715420433
nr4a1	Nuclear receptor subfamily 4 group A member 1	1	XP_004070930.	5.448553828
nr2f6	Nuclear receptor subfamily 2 group F member 6	0.99965987	XP_004078947.	2.972767361
nr1d2	Nuclear receptor subfamily 1 group D member 2	0.99849059	XP_011492490.	2.150889227
nanos3	Nanos homolog 3	0.99946184	NP_001116300.	4.778718695
lef1	Lymphoid enhancer-binding factor 1	0.99665026	XP_011474499.	4.026472211
kdm6b	lysine-specific demethylase 6B-like	0.99881383	XP_011482001.	2.468659977
kdm6a	lysine-specific demethylase 6A isoform X1	1	XP_020568543.	2.953526973
kat6b	Histone acetyltransferase KAT6B	0.99368946	XP_020561845.	3.36923381
hdac7	Histone deacetylase 7	0.99737741	XP_004070312.	2.523867903
hdac4	Histone deacetylase 4	0.99794789	XP_011488255.	2.327989848
gper1	androgen receptor alpha	0.98747173	XP_004071380.	2.266786541
gon3	Progonadoliberin-3	0.99864504	NP_001098142.	8.537747862
gnrr1	gonadotropin-releasing hormone receptor 1	0.99488797	NP_001098352.	3.101719921
gdf9	Growth/differentiation factor 9	1	XP_004073386.	4.092295995
fzd9	Frizzled-9	0.99999877	XP_004075813.	3.723658827
fzd7	Frizzled-7	0.99142416	XP_018521153.	2.060473859
fxp1b	Forkhead box protein P1-B	0.99511063	XP_020558754.	2.643653692
fstl4	Follistatin-related protein 4	0.99974672	XP_022046629.	6.242493627
foxl2	Forkhead box protein L2	1	BAH05020.1	10.0247703
foxk2	Forkhead box protein K2	1	XP_004080716.	3.740955396
foxh1	Forkhead box protein H1	0.99984307	NP_001153943.	3.971122923
foxf1	Forkhead box protein F1	0.99117670	XP_004067264.	2.300917602
figla	factor in the germline alpha isoform X1	1	XP_011477803.	5.814649649
fgfr2	Fibroblast growth factor receptor 2	0.99472363	XP_011483053.	2.046820857
fgfr1a	Fibroblast growth factor receptor 1-A	0.99999999	BAF49180.1	2.187471297
fgf3	Fibroblast growth factor 3	0.99945322	XP_011485317.	5.856066337
fgf16	Fibroblast growth factor 16	0.99945682	XP_004073393.	4.962267353
fgf13	Fibroblast growth factor 13	0.99511659	XP_011478253.	3.532269607
fgf12	Fibroblast growth factor 12	0.99952503	XP_020556550.	3.398233681
dvl1	Segment polarity protein dishevelled homolog DVL-1	0.99968349	XP_008276717.	2.612405897
dhbp7	5.6e-122/3-keto-steroid reductase	0.99951301	XP_004068242.	2.63599101
cp3ar	Cytochrome P450 3A27	0.99999997	XP_004077330.	2.413655224
cp2k1	Cytochrome P450 2K1	0.99999999	AGN04293.1	3.747955999

cp26a	cytochrome P450, family 26, subfamily A,	1	NP_001265772.	6.845619377
cp24a	1,25-dihydroxyvitamin D(3) 24-hydroxylase	1	XP_020558883.	6.074992301
cp19b	cytochrome P450 19b	0.99949942	XP_020559313.	5.466978428
cp19a	cytochrome P450 19A1-like	1	NP_001265808.	11.51191734
cp191b	cytochrome P450 19b	0.99949942	XP_020559313.	5.466978428
bmr1b	Bone morphogenetic protein receptor type-1B	0.99766148	XP_020561516.	2.259910442
bmp7	Bone morphogenetic protein 7	0.99809437	XP_004070841.	2.184590389
bmp15	Bone morphogenetic protein 15	1	XP_004079992.	10.84999144
arb	androgen receptor beta subtype	1	BAI58984.1	4.132855278
ara	Androgen receptor alpha	0.99958649	AHF58586.1	3.936856017
